# Pediatric pituitary neuroendocrine tumors–a 13-year experience in a tertiary center

**DOI:** 10.3389/fonc.2023.1270958

**Published:** 2023-11-07

**Authors:** Xiaoxu Li, Kan Deng, Yi Zhang, Ming Feng, Bing Xing, Wei Lian, Yong Yao

**Affiliations:** Department of Neurosurgery, Peking Union Medical College Hospital, Chinese Academy of Medical Sciences and Peking Union Medical College, Beijing, China

**Keywords:** pediatric, pituitary neuroendocrine tumor, surgery, invasion, prognosis

## Abstract

**Introduction:**

Pediatric pituitary neuroendocrine tumor is a rare condition, and despite previous research focusing on this specific group, the main factors influencing the surgical cure rate have not been identified.

**Methods:**

We conducted a single-center retrospective study on pediatric pituitary neuroendocrine tumor patients who visited Peking Union Medical College Hospital between 2010 and 2023. We collected data on their clinical characteristics, imaging features, surgical outcomes, and follow-up information. Additionally, we used multiple-factor logistic regression to investigate the factors affecting the surgical cure rate of pediatric pituitary neuroendocrine tumor.

**Results:**

232 patients were diagnosed with pediatric pituitary neuroendocrine tumors, with a higher incidence in females. The most common type was ACTH-secreting adenoma (90/232), followed by prolactin-secreting adenoma (63/232), and growth hormone-secreting adenoma (41/232). The majority of pediatric adenomas were macroadenomas (139/232), and some tumors were associated with cystic changes or hemorrhage (58/232), while a few exhibited invasion of the cavernous sinus (33/232). The results of the multivariate analysis indicated that the different hormone secretion types, macroadenoma or the presence of cystic changes or hemorrhage were not significant risk factors for the cure rate after the first surgery. However, the invasion of the cavernous sinus was found to be an important factor influencing the postoperative cure rate. Most pediatric pituitary neuroendocrine tumors with cavernous sinus invasion were macroadenomas, and some displayed characteristics of refractory pituitary neuroendocrine tumors, with some patients experiencing irreversible complications after surgery.

**Conclusion:**

Pediatric pituitary neuroendocrine tumors are complex, and the postoperative cure rate is particularly poor for tumors with cavernous sinus invasion. Although macroadenoma itself does not significantly impact the postoperative cure rate, it is still recommended to diagnose and treat early to avoid unnecessary surgery or surgical complications.

## Introduction

Pituitary neuroendocrine tumor (PitNET) is a pituitary disorder, accounting for approximately 15% of intracranial tumors (1). With increasing awareness and improved detection methods, the incidence of pituitary neuroendocrine tumors has risen to 115 cases per 100,000 people (2, 3). However, pediatric pituitary neuroendocrine tumors (PPitNETs) are extremely rare (4, 5), accounting for approximately 3% of all intracranial neoplasms in children and 2-6% of surgically treated PPitNETs (6–11).

In recent years, an increasing number of studies have focused on PPitNETs (12, 13). A study conducted at Mayo Clinic included 39 surgically confirmed PPitNETs reported a surgical cure rate of 46% (14). The study from Weill Cornell Medicine included 11 pediatric patients with PitNETs and indicated that compared to adult PitNETs, PPitNETs are more aggressive, exhibit higher hormone secretion, and are more difficult to treat (15). Therefore, in this study, we conducted a retrospective analysis of 232 patients diagnosed with PPitNETs in Peking Union Medical College Hospital (PUMCH), which is one of the biggest pituitary centers in China, and our aim was to contribute to the understanding of PPitNETs through this comprehensive investigation.

## Materials and methods

### Patients’ collection

We conducted a screening of patients who underwent neurosurgery at PUMCH between January 2010 and January 2023. The inclusion criteria for this study were: 1. Patients who underwent surgery and had their lesions pathologically confirmed as PitNETs. 2. Patients who were under 18 years of age at the time of diagnosis. The exclusion criteria for this study were: 1. Patients who were not diagnosed with PitNETs or had ambiguous diagnoses. 2. Patients who did not undergo surgery. This study has been reviewed and approved by the Ethics Committee of PUMCH, and informed consent forms have been signed by all patients and their guardians.

### Clinical data collection

In this study, the following clinical information of the patients was specifically collected: age, sex, duration of illness before admission, clinical symptoms and signs, history of medication or radiation therapy for PitNETs, type of medication used and its effectiveness, surgical approach and perioperative management, the need for further medication or radiation therapy after initial surgery, and whether there was tumor progression requiring repeat surgery during follow-up. The preoperative or postoperative follow-up endocrine evaluation includes not only the measurement of systemic pituitary-related hormones such as plasma cortisol, adrenocorticotropic hormone (ACTH), growth hormone (GH), prolactin (PRL), insulin-like growth factor 1 (IGF-1), thyrotropin (TSH) Triiodothyronine (T3), Thyroxine (T4), free tetraiodothyronine (FT4), free triiodothyronine (FT3), luteinizing hormone (LH), follicle-stimulating hormone (FSH), estradiol, testosterone, and 24-hour urinary free cortisol, but also the assessment of endocrine function through various endocrine tests. These tests may include the oral glucose tolerance test, the low-dose and high-dose dexamethasone suppression Test, bilateral inferior petrosal sinus sampling with desmopressin stimulation test, and the growth hormone suppression test, among others, to provide further clarification and confirmation. Due to the potential stalk effect, PRL > 200 ng/ml is considered indicative of PRL PitNET or a multiple hormone secretion tumor with PRL secretion.

### Imaging data collection

The diagnosis of PPitNETs is complex, and during the diagnostic and treatment process, multiple examinations are often required to gain further understanding of the patient’s condition. These examinations may include bone age assessment to evaluate pediatric growth and skeletal maturation. However, all patients need to undergo preoperative dynamic contrast-enhanced Magnetic Resonance Imaging (MRI) of the pituitary. In our study, two qualified radiologists collected the radiological information of the tumors based on the imaging findings in T1-weighted, T2-weighted, and contrast-enhanced T1-weighted images, and the final results were reviewed by a supervising physician. We specifically focused on the location of the tumor, the maximum diameter of the tumor, the knosp classification of the tumor, and the presence of cystic changes or apoplexy in the tumor (16). It is worth noting that some tumors, especially microadenomas in CD, may not have specific findings on pituitary MRI despite clinical and endocrine evidence. In such cases, further evaluation of the lesions was performed using Positron Emission Tomography/Computed Tomography (PET/CT) or PET/MRI.

### Data analysis

Continuous data were summarized using means ± standard deviations (SDs) or a range from the minimum to the maximum value, while categorical data were summarized using frequencies and percentages. The chi-squared test was used to compare categorical variables. Student’s t-test was employed to assess differences between normally distributed continuous variables, while the Mann-Whitney U test was used for variables that did not follow a normal distribution. Using multiple logistic regression to study the effects of multiple independent variables on a binary dependent variable. Statistical significance was considered when the p-value was less than 0.05. Data analysis was performed using SPSS (version 26.0, IBM, USA), and GraphPad Prism 10 (GraphPad Software, Inc., La Jolla, CA, USA).

## Results

### Demographics and classification

A total of 232 PPitNETs patients met the inclusion criteria and were assessed in our study. There were 128 female (55.2%) and 104 (44.8%) male patients. The median age at diagnosis was 15 years. The median duration of the pre-hospital course for PPitNETs was 2 years (0.73, 3.00). Of the 232 reported PPitNETs, 204 (87.9%) were functioning adenomas, but 28 (12.1%) were non-functioning NF adenomas. Based on the type of hormone secretion, the majority of PPitNETs patients were classified as CD (90/232), followed by PRL PitNETs accounting for 63/232 and GH PitNETs accounting for 41/232. Relatively less common types were tumors with multiple hormone secretions, which accounted for only 8/232 cases, and TSH PitNETs accounted for 2/232 cases. There are differences in the sex distribution among different subtypes of PPitNETs. CD and PRL PitNETs are more commonly seen in females, while GH PitNETs and NF PitNETs are more commonly seen in males. Clinical information of PPitNETs is shown in **Table 1**.

**Table 1:** Clinical characteristics and surgical outcomes of PPitNETs.

Hormone secretion types	ACTH	GH	PRL	TSH	NF	multiple
**n**	90,	41,	63,	2,	28,	8,
**age, years**	14.9 ± 3.0	14.8 ± 3.6	15.7 ± 1.9	8.5 ± 2.1	14.5 ± 2.4	13.5 ± 4.6
**sex, female**	52, 57.8%	13, 31.7%	48, 76.2%	2, 100%	9, 32.1%	4, 50%
**headache**	23, 25.5%	17, 41.5%	31, 49.2%	1, 50%	15, 53.6%	3, 37.5%
**visual impairments**	20, 22.2%	16, 39.0%	21, 33.3%	0, 0	14, 50.0%	4, 50.0%
**pituitary hypofunction**	13, 14.4%	4, 9.8%	2, 3.2%	0, 0	6, 21.4%	3, 37.5%
**macroadenoma**	10, 11.1%	37, 90.2	55, 87.3%	1, 50%	28, 100%	8, 100%
**CS invasiveness**	8, 8.9%	4, 9.8%	12, 19.0%	1, 50%	4, 14.3%	4, 50%
**cystic change or apoplexy**	1, 1.1%	12, 29.3%	30, 47.6%	0, 0	13, 46.4%	2, 25%
Ki-67 proliferation index *	<1% - 20%	<1%-5%	1%-10%	<1%	<1%-10%	1%-2%
**length of follow-up, years**	0.9-13	0.9-13	0.9-13	7-9	0.8-13	5-13
**disease cured after initial surgery**	62, 68.9%	23, 56.1%	44, 69.8%	1, 50%	21, 75%	1, 12.5%
**recurrent/persistent disease after initial surgery**	26, 28.9%	17, 41.5%	19, 30.2%	1, 50%	7, 25%	7, 87.5%
**any repeat surgery**	23, 25.6%	15, 36.6%	2, 3.2%	0, 0	6, 21.4%	2, 25%
**any postoperative radiation or pharmacotherapy**	9, 10.0%	4, 9.8%	17, 27.0%	1, 50%	2, 7.1%	4, 50%

*In this section, only 162 available tumor data are presented.

### Clinical presentations of PPitNETs

In addition to the specific clinical manifestations caused by high hormone secretion levels in different subtypes of pituitary tumors, such as the round red face seen in CD, acromegaly and gigantism observed in GH PitNETs, we found a significant number of patients presenting with headaches (90/232), visual impairments (75/232), and pituitary hypofunction (28/232).

### Radiological features of PPitNETs

All patients underwent preoperative pituitary MRI examinations, and based on the size of the tumors, they were classified as microadenomas and macroadenomas. Microadenomas refer to tumors with a maximum diameter smaller than 1 cm, while macroadenomas are defined as tumors with a maximum diameter equal to or larger than 1 cm. In our study, 139/232 (59.9%) had macroadenomas and 93/232 (40.1%) harbored microadenomas. In addition to CD, which mainly presents as microadenomas (80/90), GH PitNET, PRL PitNET, NF PitNET, and tumors with multiple hormone secretions are primarily characterized as macroadenomas. There was one macroadenoma and one microadenoma in TSH tumors. Additionally, knosp grade 3 and grade 4 are considered indicative of cavernous sinus(CS) invasion (17). In our study, 33 patients exhibited signs of CS invasion. Among the 232 patients, 58 experienced cystic changes or apoplexy in thePitNET. Among these patients, 30 had PRL PitNETs, 13 had NF PitNETs, 12 had GH PitNETs, 2 had tumors with multiple hormone secretions, and 1 patient with CD presented with tumor cystic changes.

### Therapies

In our study, 77/232 patients required further treatment after the initial surgery due to persistent symptoms or tumor recurrence. This included additional surgeries, radiation therapy, and medical treatments such as cabergoline, bromocriptine or temozolomide. Some patients may have received more than one type of further treatment. Among 33 patients with PPitNETs invading CS, 8 were ACTH PitNETs patients, accounting for 8/90 of the total ACTH PitNETs population. All 8 patients received subsequent radiotherapy after the first surgery. Among them, 3 patients underwent treatment with temozolomide following the radiotherapy. Among the 12 patients with prolactinomas exhibiting CS invasion, 4 patients required additional radiation therapy due to poor response to medication after surgery, and 8 patients continued to receive cabergoline or bromocriptine treatment after surgical intervention for compression relief. Additionally, among these 33 patients with invasive tumors, there were 4 patients with GH PitNET, 4 patients with tumors showing multiple hormone secretion, 4 patients with NF PitNET, and 1 patient with TSH PitNET. Among 4 patients with GH PitNET exhibiting CS invasion, 3 patients underwent repeat surgeries. As for patients with CS invasive mixed tumors, all received either radiotherapy or medical treatment after the initial surgery. Among 4 patients with CS invasive NF PitNET, two underwent repeat surgery, and two received post-operative radiotherapy. Additionally, a child with a CS invasive TSH PitNET also received further radiotherapy after surgery.

### Multiple logistic regression

**Figure 1:**
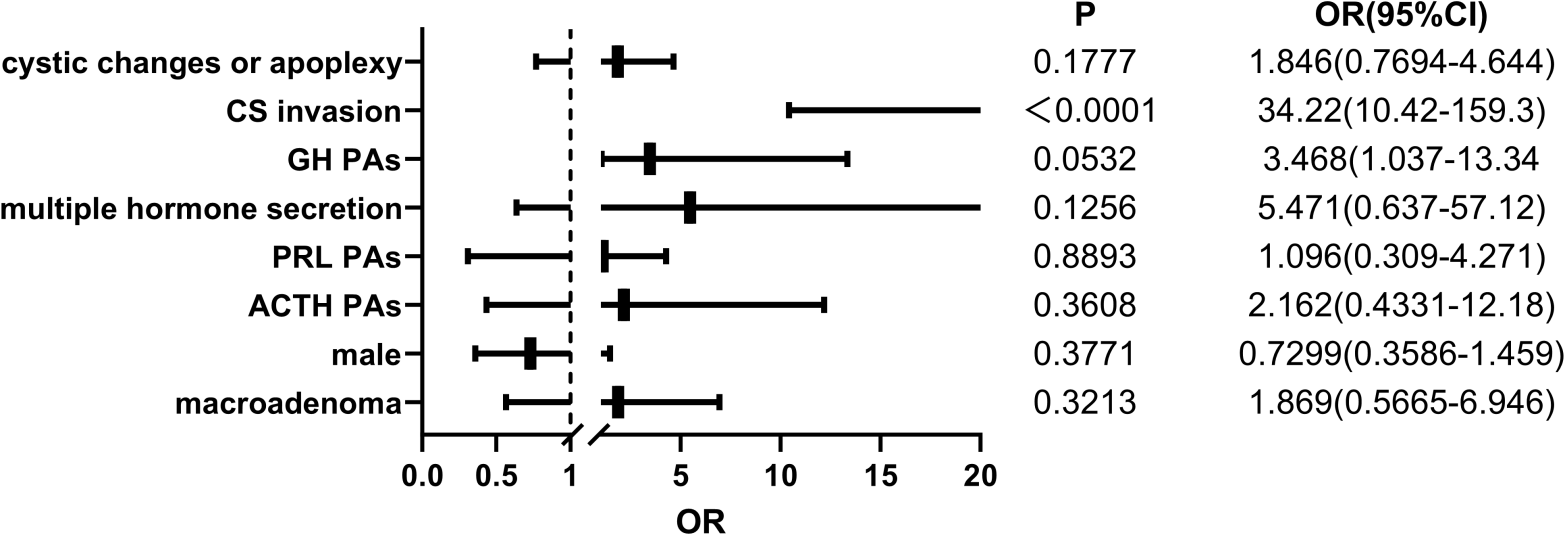
The logistic regression results of 232 pediatric pituitary adenoma patients regarding potential risk factors associated with non-remission or recurrence after surgery. OR, odds ratio. 95% CI, 95% confidence interval.

To investigate the factors that may influence the initial postoperative cure rate of patients, we conducted a multiple logistic regression analysis and calculated the odds ratio, excluding two TSH tumor samples due to a small sample size. The condition is only considered cured during a follow-up period of at least greater than six months when postoperative follow-up MRI shows no residual tumor, and the patient no longer exhibits excessive hormone secretion. The variables included in the analysis were patients’ sex, tumor hormone secretion type, tumor size, presence of CS invasion, and presence of cystic changes or apoplexy. The results of the multiple factor analysis showed no difference in the rate of non-remission or recurrence after initial surgery between sexes. The excessive secretion of ACTH, PRL, or GH, compared to NF PitNETs, did not increase the risk of tumor persistence or recurrence. Furthermore, patients with macroadenomas did not show an increased risk compared to those with microadenomas. Additionally, tumors with cystic changes or apoplexy did not exhibit a higher relative risk. In our study, the significant risk factor affecting non-remission or recurrence was the invasion of the tumor into the CS (p<0.05) (**Figure 1**).

## Discussion

In recent years, there has been increasing research focus on pediatric patients with pituitary diseases. Similar to previous studies, PPitNETs in our research predominantly manifested as functional tumors and macroadenomas, and were predominantly seen in female (14, 18, 19). In contrast to some previous single-center studies, where PRL PitNET or GH PitNET were reported as the two most common types (6, 20), however, in our study, CD was the most common type of PPitNETs, surpassing PRL PitNET, which is consistent with the findings of a large-scale systematic review reported earlier (14). This may be related to the fact that some PRL PitNET patients can achieve good therapeutic effects through medication such as cabergoline and bromocriptine (21–24), thus avoiding the need for surgery. Although previous studies have provided detailed descriptions of the clinical presentations of different types of PPitNETs, there is still limited research reporting whether there are differences in the non-remission or recurrence rates after surgery among different types of PPitNETs, as well as the imaging features that may provide us with clues to the risk factors contributing to persistent/recurrent conditions. In our study, we found that although PPitNETs were predominantly functional tumors, the coexistence of high hormone secretion did not increase the probability of non-remission and recurrence after initial surgery for patients. Given that a considerable number of patients experienced headaches, visual impairment, and even pituitary hypofunction, we focused on the influence of tumor size and the presence of cystic changes or apoplexy on the rate of surgical non-remission or recurrence. While tumors with cystic changes or apoplexy can cause visual impairment due to mass effect on the optic chiasm and pituitary hypofunction due to compression of the normal pituitary (25, 26), we did not find statistically significant differences in the rate of non-remission and recurrence after initial surgery in patients with the presence of them. However, although tumors invading CS are not predominant in PPitNETs (33/232), the results of multiple regression analysis showed that aggressive tumors invading CS had a poorer rate of permanent remission after initial surgery compared to tumors without presence of CS invasion. Therefore, we will now focus on discussing PPitNETs invading CS in more detail.

ACTH PPitNETs are worthy of attention. ACTH PitNETs can lead to elevated cortisol levels by excessive ACTH secretion, which affects the adrenal glands (27). For pediatric patients, the high cortisol levels can not only cause pathological changes commonly seen in adults, such as impaired glucose metabolism, lipid disorders, and hypertension, but also significantly interfere with the child’s growth in height (28–30). On one hand, the elevation of blood cortisol levels can lead to growth retardation in children since the onset of the disease. On the other hand, pediatric CD can lead to the development of osteoporosis (31), and osteoporosis can cause vertebral compression, affecting their height. Therefore, if not treated promptly, even after treatment, there may not be significant growth in height. Although TSS is the preferred treatment for CD (27, 32, 33), in our cases of CD PitNET with CS invasion, there is no patients achieved complete tumor resection and restoration of endocrine hormone levels through a single surgery, and all of them required subsequent radiation therapy. Stereotactic radiotherapy has been proven to be effective for approximately 92% of patients with residual ACTH-secreting pituitary neuroendocrine tumors that cannot be relieved through surgery (34, 35). However, in our case series, a significant proportion of patients still exhibited insensitivity to radiotherapy and therefore received treatment with temozolomide. Despite several studies indicating that temozolomide can induce tumor shrinkage and restore endocrine hormone levels in PitNETs (36–39), one patient in our study showed disease progression even after receiving 3 sessions of gamma knife and 9 cycles of temozolomide chemotherapy. Whole-genome sequencing indicated the presence of germline mutations in G protein-coupled receptor 101 (GPR101) and somatic mutations in Ubiquitin Specific Peptidase 8 (USP8) in this child, which were not present in her parents. USP8 mutations have been identified as common genetic alterations in CD patients, which can activate the epidermal growth factor receptor (EGFR) signaling pathway and promote the development of CD (40). In a study focused on pediatric patients with ACTH PitNETs, USP8 mutations were found in 31% of cases. However, USP8 mutation carriers generally have small tumor size, which is no more than 5 mm (41). In this case, the preoperative dynamic contrast-enhanced MRI of the patient revealed a tumor size of 51mm, with upward extension into the suprasellar cistern, depression of the sellar floor bone, significant compression and elevation of the optic chiasm, and bilateral CS invasion, classified as knosp grade 4, displaying a diffuse and invasive growth pattern. An isolated USP8 mutation alone cannot fully explain the entire behavior of the tumor. Studies have shown that GPR101 mutations can lead to X-linked acrogigantism (X-LAG) syndrome (42), a familial or sporadic pituitary disorder caused by chromosome Xq26.3 microduplications (43). The syndrome is characterized by excessive GH and IGF-1 levels, resulting in abnormal rapid growth even within a few months after birth (43). Although GPR101 has been confirmed to promote GH hypersecretion and gigantism through the Gs and Gq/11 signaling pathways in mice (44, 45), its involvement in the pathogenesis of CD remains uncertain.

Although in our study, macroadenomas or tumors with cystic changes or apoplexy, did not pose a threat to the surgical cure rate, it does not imply that these factors are unimportant. In our study, prolactinomas with CS invasion presented as macroadenomas, and nearly half of these tumors exhibited cystic changes or apoplexy. Therefore, some patients required surgical intervention for timely decompression, even though their prolactinomas might be responsive to medication and not necessarily require surgery. It is worth mentioning that one patient with a prolactinoma, despite exhibiting a favorable response to bromocriptine in preoperative drug trials, experienced cerebrospinal fluid (CSF) leakage shortly after medication due to the extensive tumor invasion into the sellar floor bone. To prevent the exacerbation of CSF leakage caused by further tumor shrinkage after medication and the subsequent development of retrograde intracranial infection, we had to temporarily suspend drug therapy and perform both pituitary neuroendocrine tumor resection and CSF leak repair surgery. Therefore, early identification of PPitNETs and timely intervention may be necessary, as large tumor burden is not always completely resolved through surgery and may even lead to unnecessary surgical intervention in patients who are responsive to medication.

## Limitations

Our study has several limitations. Firstly, due to its retrospective nature and incomplete data in some cases, we did not include genetic causes and certain pathological indicators in the multifactorial regression analysis in this article. Secondly, despite a relatively large number of cases, this study is still a single-center study. Therefore, further research on PPitNET is still needed in the future.

## Conclusion

In conclusion, our study provides a comprehensive report on a large series of patients with PPitNET, collecting and presenting clinical characteristics, imaging features, surgical outcomes, and follow-up data. Through multivariate regression analysis, we found that the presence of hormone secretion did not affect the initial surgical cure rate of patients. Importantly, the presence of CS invasion significantly influenced the initial surgical cure rate. In our PPitNET cohort, we found that the majority of tumors with CS invasion were macroadenomas. Although in our study, macroadenoma itself was not identified as a risk factor for symptom persistence or recurrence after tumor resection, early diagnosis and intervention may still be necessary, as some complications from macroadenoma surgery can have a significant impact on children.

## Data availability statement

The original contributions presented in the study are included in the article. Further inquiries can be directed to the corresponding author.

## Ethics statement

The studies involving humans were approved by the Ethics Committee of Peking Union Medical College Hospital. The studies were conducted in accordance with the local legislation and institutional requirements. Written informed consent for participation in this study was provided by the participants’ legal guardians.

## Author contributions

XL: Conceptualization, Formal Analysis, Writing – original draft. KD: Formal Analysis, Writing – original draft. YZ: Formal Analysis, Writing – original draft. MF: Investigation, Writing – review & editing. BX: Investigation, Writing – review & editing. WL: Methodology, Writing – review & editing. YY: Conceptualization, Funding acquisition, Methodology, Writing – review & editing.
